# Personality and Injury Risk Among Professional Hockey Players

**DOI:** 10.5249/jivr.v1i1.8

**Published:** 2009-07

**Authors:** Zachary H Osborn, Paul D Blanton, David C Schwebel

**Affiliations:** ^a^University of Alabama at Birmingham, USA.

## Abstract

**Background:**

Although much is known about risk for athletic injury, research on the roles of individual differences in personality and temperament on athletic injury has lagged. We hypothesized that professional athletes with high sensation-seeking and extraversion scores, and with low effortful control scores, would experience more injuries over the course of a season, would have more severe injuries, and would miss more total days of play.

**Methods:**

Prospective design with questionnaire report at time one and injury tracking throughout an 18-week athletic season. Setting: Professional hockey team in the United States. Participants: Eighteen professional hockey players (ages 21-33).

**Measurement:**

Players completed self-report personality (Sensation-Seeking Scale, Form V) and temperament (the Adult Temperament Questionnaire) measures. Quantity and severity of injury, as well as playing time missed, were tracked for 18 weeks.

**Results:**

On average, players experienced almost 6 injuries causing a loss of 10 playing days through the season. Those players scoring high on Boredom Susceptibility and Total Sensation-Seeking incurred more total injuries. Those scoring high on temperamental neutral perceptual sensitivity suffered more severe injuries.

**Conclusions:**

Athletes who suffered more injuries reported a preference for stimulating environments and boredom with non-stimulating environments. Injury severity was not correlated with sensation-seeking but was related to temperamental perceptual sensitivity. Implications for identification of injury-prone athletes, pre-injury training, and post-injury treatment are discussed.

## Introduction

At some point in their careers, almost all professional athletes - particularly those in contact sports such as ice hockey - experience injuries.^1^ Epidemiological data on injury rates among professional hockey players suggest professional hockey players experience an average of over ^2^ concussions per season2 and male college ice hockey players experience injuries at a rate of 9.19 per 1000 athlete-exposures (i.e., game, practice, or weight-training session).^3^

Many athletes assume injuries are truly “accidental” - that is, the result of stable and global causes that cannot be prevented or controlled,^4^ but researchers have long recognized the fallacy in those beliefs and attribute athletic injury risk to a variety of preventable risk factors, including players’ alcohol use,^5^ sleep patterns,^6^ and gender.^7^ Another factor that may contribute to injury risk among professional athletes, and the focus of the current investigation, is the athletes’ individual differences in personality and temperament.

A large body of pediatric research links general injury risk to individual differences in temperament and personality.^8^ Children scoring high in temperamental traits of extraversion, activity level, impulsivity, and undercontrol have increased risk of both minor daily injuries^9^ and major injuries requiring professional medical attention.^10-11^ Parallel results have been reported in the adult literature.^12-15^ In a retrospective study of spinal cord injured and non-injured persons, for example, injured individuals had higher sensation-seeking scores than their non-injured counterparts.^14^ A study examining the difference between risky driving behaviors among head-injured and non-head-injured drivers showed that judgment of risk was similar between the two groups, but those who suffered head injuries reported a preference for high risk situations and engaged in more high-risk activities.^15^ Given the prevalence of concussions and other injuries in the sport of hockey, these findings suggest that players are likely to engage in and prefer riskier behaviors and environments, thus increasing the likelihood of serious injury.

Surprisingly, relatively little research examines links between individual differences in personality and risk for athletic rather than general injury. In one study, researchers assessed high school football players’ sensation-seeking and then tracked injuries throughout the season.^16^ Results indicated sensation-seeking did not predict injury incidence. Schwebel and colleagues reported similar findings in a study of temperament and soccer injuries among a sample of pre-adolescent male soccer players.^17^ Two studies have examined links between sensation-seeking tendencies and skiing injuries.^18-19^ Surprisingly, both reported a link between lower rates of sensation-seeking and higher rates of injury. Taken together, we might conclude there is mixed evidence on the links between personality differences and adult athletic injury. Available research is limited in sport, with a focus on skiers among adult samples. No research has studied a sample of professional athletes, and no research has examined a sample of hockey players. The studies that have been published contradict findings in the general injury literature and suggest personality may not predict athletic injury risk in adults.

The present study was designed, therefore, to investigate the role of individual personality differences in predicting athletic injuries among a sample of professional hockey players. We focused on hockey players primarily because of the high injury risk in the high-contract sport; it also is an understudied population in the literature. We hypothesized that athletes with high sensation-seeking and extraversion scores, and with low effortful control scores, would experience more injuries over the course of a season. We also hypothesized that those scoring higher in sensation-seeking and extraversion and those scoring lower in effortful control would have more severe injuries and would miss more total days of play than those with lower scores.

## Methods

**Participants**

All eighteen members on a minor-league professional hockey team participated in the study. All participants were male, Caucasian, and between the ages of 21 and 33 (M = 27.39, SD = 3.59). Seventeen of the 18 players (94%) were native English speakers and all were proficient in reading and speaking English. Players underwent comprehensive medical examinations prior to the season that cleared them as healthy to compete in the league.

**Measures**

Sensation-Seeking. Sensation seeking was assessed using Zuckerman’s Sensation-Seeking Scale, Form V (SSS-V).^20^ The SSS-V consists of four subscales derived through factor analysis that can be summed to obtain a total score. The Thrill and Adventure Seeking (TAS) subscale measures individuals’ desire to engage in risky or stimulating sports and activities. The Disinhibition (DIS) subscale reflects a general pattern of non-conformity to societal norms. Boredom Susceptibility (BS) measures the individuals’ dislike of repetitive activities. Experience Seeking (ES) measures individuals’ desire to seek stimulation through new experiences and activities such as art, travel, and drugs. The SSS-V is widely used and has good internal reliability.^20^

Temperament. Temperament was assessed using the Adult Temperament Questionnaire (ATQ),^21^ which consists of four broad factors and thirteen subscales. The Negative Affect factor (NA-ATQ) includes the subscales fear, frustration, sadness, and discomfort and assesses the individual’s level of experienced general negative feelings. Extraversion/ Surgency (ES-ATQ) includes the subscales sociability, high intensity pleasure, and positive affect and assesses the person’s affinity for high-energy, high-stimulus, social situations. Effortful Control (EC-ATQ) includes the subscales activation control, attentional control, and inhibitory control, and measures the person’s self-reported ability to control or direct their behaviors in a desired direction. Orienting Sensitivity (OS-ATQ) includes the subscales neutral perceptual sensitivity, affective perceptual sensitivity, and associative sensitivity and assesses the person’s general awareness of their surroundings. The ATQ has adequate internal reliability and validity.^21-22^

Injuries. Each player’s injuries, the number of practices and games they missed, and the treatments used for incurred injuries were tracked by the team’s athletic training staff. Total injuries were calculated by adding the number of new injuries sustained by each player for the duration of the study. The count of new injuries started from the first day of practice through the conclusion of the study. Once a player was cleared to return, any later injuries were classified as new injuries. No player had a pre-existing injury prior to the start of the study. Injury severity was computed based on intensity of treatment required from the athletic training staff and was coded on a 5-point scale (1 = ice bag or heat pack, 5 = treatment from physician). Days missed were calculated from a daily coaches report that indicated whether the player could practice, engage in limited practice, or not practice, based on health. The days missed variable was a sum of all days when the player was not released for practicing at full effort (the team either practiced or played games 5 days per week, on average).

**Procedure**

Details about all injuries incurred by players on the team were recorded by athletic training staff for the first 18 weeks of the season. Injury records were kept during both practices and games, and were then submitted to the researchers. Players completed the SSS-V and ATQ midway through the season. All research was approved by the university’s IRB and informed consent was obtained from all players. The team’s coaches, training staff, and management also consented to the research program.

## Results

Data were analyzed in two steps: (a) descriptive analyses and (b) correlational analyses. An alpha of .05 was used to establish statistical significance, but because of low statistical power (power < .30 to detect medium effect size, a= .05), findings with p < .10 are reported as trends.

**Descriptive Statistics**

Table 1 lists descriptive statistics for all variables of interest. The athletes experienced an average of 5.94 injuries through the 18-week study (SD = 4.96, range = 0 to 16). The average time lost from play (sum of practice and game days missed) was 9.61 days (SD = 21.47, range = 0 to 91) and the mean injury severity rating was 1.81 (SD = 1.16, range = 0 to 4.50). Table 1 also includes average scores for the SSS-V and ATQ scales.

**Table:** Table 1: **Descriptive and Correlational Data for Demographic, Injury, Sensation Seeking, and Temperament Variables (N = 18)**

		Correlations		
Variable	M (SD)	Total Injury	Injury Severity	Days Missed
Demographics				
Age	27.39 (3.59)	-.14	-.02	-.12
Injury Measures				
Total Injuries	5.94 (4.96)	--	.27	-.02
Average Severity	1.81 (1.16)	.27	--	.05
Days Missed	9.61 (21.47)	-.02	.05	--
Sensation Seeking				
Total Score	20.44 (6.19)	.55*	.19	.09
TAS	6.39 (2.70)	.33	.28	.06
ES	5.17 (1.98)	.40+	-.24	.25
DIS	5.83 (2.83)	.33	.13	-.02
BS	3.06 (1.66)	.47*	.31	-.01
Temperament				
Negative Affect	3.91 (0.36)	.16	.06	.14
Fear	3.92 (0.67)	.29	.24	.15
Frustration	3.97 (0.81)	-.38	.13	-.02
Sadness	3.83 (0.54)	.36	.15	.11
Discomfort	3.92 (0.77)	.19	-.33	.08
Extraversion /Surgency	3.81 (0.44)	-.17	.26	-.02
Sociability	3.96 (0.50)	-.17	.28	.06
Positive Affect	3.33 (0.82)	-.20	.28	-.05
High Intensity Pleasure	4.13 (0.85)	.03	-.03	-.02
Effortful Control	3.93 (0.51)	.00	.41+	.26
Activation Control	4.02 (0.70)	-.03	.06	.00
Inhibitory Control	3.66 (0.81)	.10	.44+	.22
Attentional Control	4.12 (0.59)	-.10	.39	.39
Orienting Sensitivity	3.88 (0.46)	-.09	-.07	.12
Neutral Perceptual Sensitivity	3.52 (0.55)	.06	.51*	.14
Affective Perceptual Sensitivity	4.38 (0.69)	.20	-.24	.20
Associative Sensitivity	3.74 (1.08)	-.06	-.20	-.04

* p ≤ .05 + p ≤ .10 Note. TAS = Thrill and Adventure Seeking; ES = Experience Seeking; DIS = Disinhibition; BS = Boredom Susceptibility

**Correlations**

Sensation-Seeking and Injury. Correlation analyses with the SSS-V suggest total injuries were significantly correlated with the Boredom Susceptibility subscale (r (16) = .47, p < .05) and the total sensation seeking score (r (16) = .55, p < .05), and approached statistical significance with the Experience Seeking (r (16) = .40, p = .10) subscale (See Table 1).

Temperament and Injury. Correlation analyses with ATQ suggested that average injury severity was significantly correlated with neutral perceptual sensitivity (r (16) = .51, p < .05) and that average injury severity approached a significant correlation with the inhibitory control subscale (r (16) = .44, p = .07) and the effortful control factor scale (r (16) = .41, p = .09; see Table 1). Total injuries incurred were not significantly correlated with any of the factors or subscales on the ATQ.

## Discussion

Athletes who suffered more injuries reported a preference for stimulating environments and boredom with non-stimulating environments.

This finding is consistent with work studying temperamental and personality predictors of general injury in both children ^8-11,23^ and adults,^12-15^ but it is inconsistent with existing work on personality predictors and athletic injury.^16-19^ The finding is theoretically sensible: sensation seeking athletes] may be more likely to take risks and place themselves in potentially dangerous situations more often than athletes who are not prone to sensation seeking. Severity of injury was not linked to sensation-seeking but a positive correlation did emerge between neutral perceptual sensitivity temperament trait and injury severity. This finding also is theoretically sensible. The trait of neutral perceptual sensitivity encompasses sensitivity to subtle changes or differences in the environment. Athletes who score high in temperamental neutral perceptual sensitivity may experience the subjective feelings of pain more readily and therefore might be more likely to perceive and report the injury as severe.

Athletes are injured for a wide range of factors. Some risks are environmental – hockey players who fail to wear protective equipment, for example, are at greatly increased risk for injury.^24^ Other risks relate to individual differences: alcohol use,^5^ sleep patterns, ^6^ and gender differences ^7^ all appear to predict athletic injury risk. Results from this study suggest individuals’ personality and temperament, and sensation-seeking in particular, also correlates to risk among for athletic injury.

**Implications and Limitations**

Identification of risk factors for injury is most useful if such knowledge can be translated into injury prevention programs. Such programs might focus on identifying athletes whose individual differences traits make them susceptible to risk-taking and potentially injurious activities. Such identification could permit coaches, trainers, and researchers to protect those most at risk. Risk-taking players could, for example, be offered or required to wear protective equipment.

Another potential application of the study’s results is for return-to-play decisions following injury. Physiologically, all athletes are at increased risk for re-injury upon returning to play; individuals high in sensation-seeking may be particularly susceptible to re-injury due to risk-taking tendencies. Trainers or coaches might delay particular players’ return to play longer because of that increased risk.

The strengths of this study are that it assessed all players on a professional hockey team, used standardized assessment instruments, and systematically captured all injuries the athletes incurred over an 18-week period. The study also had limitations. Most prominent is the small sample size, which caused low statistical power and reliance on correlations rather than regressions. A second limitation is that the sample was limited to a single team; the team may not be representative of other hockey teams or of athletes in other sports. Finally, the timing of measurements in this study was not optimal. Because personality and temperament were measured mid-season, after some of the injuries had occurred but before others had, inferences of causality must be made cautiously. The fact that personality and temperament are viewed to be relatively stable traits mitigates this limitation somewhat. Replication of the reported findings in future work using larger and more diverse samples is needed to better understand links between individual differences and injury risk among professional athletes.
